# The learning curve for endoscopic endonasal pituitary surgery: a systematic review

**DOI:** 10.1007/s10143-023-02136-8

**Published:** 2023-09-12

**Authors:** Nicholas G. Candy, Christopher Ovenden, Alistair K. Jukes, Peter-John Wormald, Alkis J. Psaltis

**Affiliations:** 1 Department of Surgery - Otolaryngology, Head and Neck Surgery, The University of Adelaide, Basil Hetzel Institute for Translational Research, Woodville South, Adelaide, Australia; 2 Department of Neurosurgery, Royal Adelaide Hospital, Adelaide, Australia

**Keywords:** Endoscopic surgery, Pituitary, Endocrine, Neurosurgery

## Abstract

Recent literature demonstrates that a learning curve exists for endoscopic pituitary surgery. However, there is significant variability in the way these studies report their outcomes. This study aims to systematically review the literature regarding outcomes for endoscopic pituitary surgery and how this may be related to a surgical learning curve. An electronic search of the databases Medline, Scopus, Embase, Web of Science and Cochrane Library databases was performed and data extracted according 2020 Preferred Reporting Items of Systematic Reviews and Meta-Analyses (PRISMA) statement. Ten articles were included in the review as they examined the following: rates of gross total resection, average operative time, CSF leak rate, visual outcomes, endocrine outcomes and how these results were influenced by surgical experience. We have demonstrated that a learning curve exists for some outcome variables for endoscopic pituitary surgery. However, there is significant heterogeneity in the current body of literature which makes clear comparisons difficult.

**Supplementary Information:**

The online version contains supplementary material available at 10.1007/s10143-023-02136-8.

## Introduction

Pituitary adenomas are benign tumours of the pituitary gland that can be classified clinically on whether they are functioning (hypersecreting pituitary hormone/s) or non-functioning adenomas. Surgical management through endoscopic endonasal transsphenoidal resection is an accepted and commonly used technique to remove these tumours, demonstrating superior rates of gross total resection than traditional microsurgical techniques [1].

Over the past few years, there have been multiple studies demonstrating that a learning curve exists for endoscopic pituitary surgery [2, 3]. However, there appears to be significant variability in the way these studies report their outcomes. This makes understanding and comparing studies challenging.

This study aims to systematically review the literature regarding outcomes for endoscopic pituitary surgery and how this may be related to a surgical learning curve.

### Literature search

A search strategy was devised according to the 2020 Preferred Reporting Items of Systematic Reviews and Meta-Analyses (PRISMA) statement [4] (refer to supplementary Figure 1). An electronic search of the databases Medline, Scopus, Embase, Web of Science and Cochrane Library databases was performed from inception until the 7th of May 2023. To identify articles investigating how outcomes for endoscopic pituitary surgery change as a surgeon gains more experience, the following search terms were applied: (pituitary OR pituitary adenoma) AND (visual OR ophthalmology OR endocrine OR hormonal OR resection OR outcome) AND (learning curve OR experience) with prior checking in the MeSH database to include synonyms.

The database search was further supplemented by a search of the reference lists of included studies as well as checking the related article function provided by each database. Titles and abstracts were screened to identify potentially relevant studies. All potentially relevant articles, or articles where it was unclear based on the abstract, were assessed by reviews of the full-text articles.

Articles were deemed eligible if they (1) recorded preoperative information regarding visual function and endocrine function; (2) examined endocrine and ophthalmological function postoperatively; (3) reported complications; (4) performed statistical analysis on outcomes after dividing patients into groups based on when surgery was performed. Studies were excluded when (1) they did not provide long-term follow-up for endocrinological outcomes; (2) a focus of the article was not to examine the learning curve for endocrine outcome; (3) if patients undergoing microscopic pituitary surgery were included; (4) if the surgical technique changed significantly during the study period.

### Data extraction

All data was reviewed independently by 2 authors (NC and CO) and discrepancies cross-checked in a consensus meeting.

The following data was obtained from the included studies: number of patients, location of surgery, time period when operations occurred, who performed the surgeries, role of skull base ENT surgeons, duration of follow-up, how the groups were divided temporally, division of functional and non-functional tumours, all available preoperative endocrine information, visual function, average operative time, postoperative CSF leak rate, endocrine outcome, visual outcome and extent of resection.

### Quality assessment

We used a modified quality assessment tool incorporating the Cochrane Collaboration tool to assess the methodological quality of the included articles [5]. The quality assessment tool (Table 1) assessed: demographic details, preoperative variables, postoperative variables, complications and learning curve. The same two authors (NC and CO) then evaluated the risk of bias in the individual articles using a modified version of the Cochrane Collaboration method (Table 2). Discrepancies were resolved after discussion and consensus amongst all authors.

**Table 1:** Quality assessment tool

Quality category	Questions	Response		
		Yes	No	Unclear
Demographic details	Is the age and gender for each surgical group defined?			
	Is the role of neurosurgery and ENT clearly defined?			
	Are the number of patients examined clearly defined?			
	Is it defined if these cases are sequential or part of a larger surgical series?			
Preoperative variables	Is the number of functional and non-functional adenomas defined?			
	Are the different types of functional adenomas defined?			
	Is the endocrine function of non-functional adenomas clearly defined?			
	Is the method of quantitative visual assessment defined for all patients?			
	Is tumour size and tumour invasion clearly defined?			
	Is the method of assessing endocrine function clearly defined?			
Postoperative variables	Is the rate of gross total resection clearly defined for all groups?			
	Is the timing interval for when each outcome is examined clearly defined?			
	Is the method of determining visual outcome clearly defined?			
	Is the rate of endocrine cure clearly defined for all surgical groups?			
	Are the hormonal outcomes for non-functional adenomas clearly defined?			
	Is the method of how endocrine cure is determined clearly defined?			
	Is the further treatments required during follow-up defined?			
Complications	Is the rate of postoperative CSF leak clearly defined for all groups?			
	Is the rate of permanent diabetes insipidus clearly defined for all groups?			
Learning curve	Is the method for how each surgical group is created clearly defined?			
	Are all outcomes examined to see how they are affected by surgical experience?			
	Is the statistical method for how the learning curve is assessed clearly defined?

**Table 2:** Grading of quality assessment

Quality category	Poor	Moderate	Good
Demographic details	<4 criteria	3 of 4 criteria	4 of 4 criteria
Preoperative variables	<5 criteria	5 of 6 criteria	6 of 6 criteria
Postoperative variables	<6 criteria	6 of 7 criteria	7 of 7 criteria
Complications	<2 criteria		2 of 2 criteria
Learning curve	<3 criteria		3 of 3 criteria

## Results

### Study selection

**Fig. 1:**
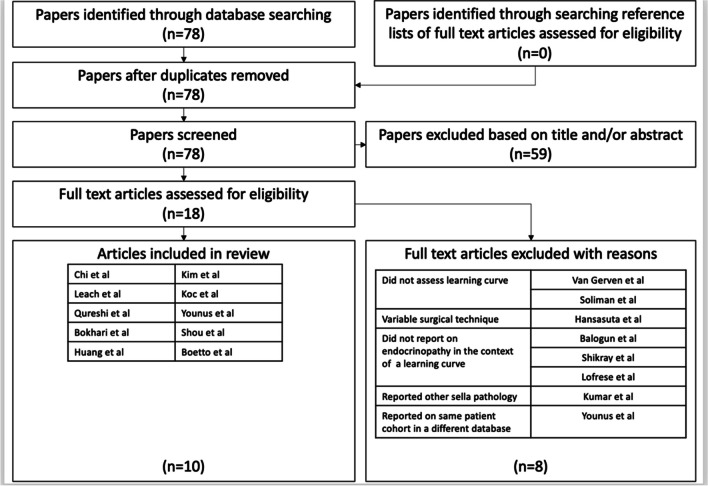
PRISMA flowchart demonstrating the results of study selection

From the literature search, 78 articles were identified through searching Medline, Scopus, Embase, Web of Science and Cochrane Library databases (refer to Fig. 1). Fifty-nine were initially excluded based on the content of the title or the abstract. The most common reason for exclusion was being unrelated to assessing the learning curve. Eighteen articles [2, 3, 6–21] proceeded to full-text review with 10 articles [2, 3, 7–9, 11, 12, 15, 20] being selected for inclusion. Of the 8 articles excluded, 2 articles [18, 19] were excluded because they did not assess the operative learning curve, 3 articles [6, 14, 16] did not report on the rate of endocrinopathy in a way that it could be examined in the context of a learning curve, 1 article [21] reports on the same group of patients within a larger database, 1 article [13] was excluded as it reported other sella pathology and 1 article [10] was excluded because the surgical technique changed significantly during the study period.

### Study characteristics

Of the 10 included studies, there was significant variation in the number of patients, duration of follow-up, methodology and method of reporting for outcome variables.

**Kenan et al.** [2] reported on 78 patients who underwent endoscopic endonasal transsphenoidal resection of a pituitary adenoma at Kocaeli University Hospital in Turkey between 1997 and 2005. Operations were performed by one of the two senior authors initially with otolaryngology assistance, but later without. The minimum duration of follow-up was 6 months with variable follow-up ranging from 44.8 months to 9 months. The groups were divided into an early group of 9 cases from 1997 to 2000 and a late group of 69 cases from 2001 to 2005. Endocrine outcomes for cure were defined for prolactinomas and somatotropinomas with other functional adenomas being excluded; patients with non-functioning adenomas did not receive assessment. Variables reported included operative time, postoperative cerebrospinal fluid (CSF) leak rate, rate of diabetes insipidus (DI), degree of resection on MRI scan at 6 months, formal visual assessment at 1 month and endocrine assessment at an undefined time interval.

**Leach et al.** [3] reported on the first 125 patients who underwent endoscopic endonasal transsphenoidal surgery at the Department of Neurosurgery Royal Salford Hospital in Manchester between 2005 and 2007. All procedures were performed by one surgeon who had been previously performing microscopic transnasal surgery for pituitary adenoma resection and had received 6 months of training in endoscopic pituitary surgery prior. The mean duration of follow-up was 18 months. The groups were divided into two 15-month periods of 53 patients from April 2005 to June 2006 and 72 patients from July 2006 to September 2007. Endocrine outcomes were defined for non-functioning and functioning adenomas. Variables reported included operative time, postoperative CSF leak rate, rate of DI, degree of resection on magnetic resonance imaging (MRI) at 4–6 months, basal and reserve pituitary function at 2 to 3 weeks and ophthalmological assessment at 4 to 6 weeks.

**Bokhari et al.** [8] reported on 79 consecutive patients who underwent endoscopic endonasal transsphenoidal resection of a pituitary adenoma at St. George Public and Private Hospitals between July 1998 and September 2010. All procedures were performed by a single neurosurgeon. The mean follow-up for patients was 38 months. Patients were divided into 3 equal groups of 27, 26 and 26 cases respectively. These groups spanned between the years 1998–2004, 2004–2006 and 2006–2010. The endocrinological cure for functional adenomas is clearly defined, but the assessment for non-functional adenomas is not clear. Variables reported included postoperative CSF leak rate, rate of DI, degree of resection on MRI scan, formal visual assessment and endocrine assessment.

**Chi et al.** [9] reported on 80 consecutive patients who underwent endoscopic endonasal transsphenoidal resection of pituitary adenoma at the Department of Neurosurgery Renji Hospital Shanghai between 2011 and 2012. All procedures were performed by a neurosurgeon without otolaryngology assistance who previously performed microscopic transsphenoidal procedures and spent 3 months training in endoscopic techniques. The duration of follow-up is undefined, but all patients had at least 1 month of follow-up. Patients were divided into an early group of patients 1 to 40, and a late group of patients 41 to 80. Endocrine outcomes for functional adenomas are defined, but non-functioning adenomas are not assessed. Variables reported include operative time, postoperative CSF leak rate, rate of DI, extent of resection on MRI, formal visual field assessment and endocrine assessment.

**Shou et al.** [17] reported on 178 consecutive patients from March 2011 to May 2014 who underwent endoscopic endonasal transsphenoidal pituitary adenoma resection at the Department of Neurosurgery, Shanghai Pituitary Tumour Centre. All procedures were performed by 2 neurosurgeons without otolaryngology assistance. All patients had at least 12 months of follow-up. Patients are divided into 2 groups with 89 patients in each group. It is unclear over what time these 2 groups encompass. Endocrine outcomes for functional adenomas are defined for somatotropinomas and prolactinomas, but not for corticotropinomas or non-functional adenomas. Variables reported included rates of resection on MRI scan, postoperative CSF leak, rate of DI and endocrine outcomes. Visual outcome is not reported.

**Qureshi et al.** [15] reported on 78 consecutive patients who underwent endoscopic endonasal transsphenoidal surgery for resection of pituitary adenoma at the Department of Neurosurgery Rush University Chicago, between 2006 and 2012. All procedures were performed by a single team of neurosurgeons and otolaryngologists. Patients had at least 6 weeks of follow-up. Patients were divided into an early group of 9 patients and a late group of 69 patients based on post hoc analysis. Endocrine outcomes for functional cure are not defined and assessment of non-functioning adenomas is not defined. Variables reported included operative time, rate of postoperative CSF leak, rate of DI, degree of resection on MRI scan, visual field and endocrine outcomes.

**Kim et al.** [12] reported on 331 patients who underwent endoscopic endonasal transsphenoidal resection of non-functioning pituitary adenomas at Seoul National University Hospital from 2010 to 2016. Operations were performed by a single surgeon. Post hoc analysis using receiver operating characteristic curve analysis was performed to determine the number of surgical cases for a difference in gross total resection. This created 2 groups: 0–100 cases between 2010 and 2011 and 101–331 cases between 2012 and 2016. Endocrine outcomes for non-functioning adenomas were defined at a specified time interval. Functional adenomas and apoplexy were excluded. Variables reported included rate of postoperative CSF leak, rate of DI, degree of resection on MRI scan, endocrine assessment and visual assessment.

**Younus et al.** [20] reported on 600 consecutive patients who underwent endoscopic transsphenoidal resection of a pituitary adenoma at the Department of Neurosurgery Will Cornell Medicine, New York, between 2004 and 2018. All surgeries were performed by a single neurosurgeon with assistance from an otolaryngologist and a neurosurgery resident or fellow. Patients were divided into 4 quartiles with 150 patients in each. Endocrine outcomes for functional adenomas are defined at a specified time interval, whereas non-functional adenomas are not defined. Variables reported included rate of postoperative CSF leak, rate of DI, degree of resection on MRI scan, endocrine assessment and visual assessment.

**Boetto et al.** [7] reported on 53 patients who underwent endoscopic transsphenoidal resection of a non-functioning pituitary adenoma between November 2017 and November 2020 at the Department of Neurosurgery, Montpellier University Medical Centre. Cases were performed by a single neurosurgeon with ENT assistance. Patients had a minimum of 12 months follow-up. Patients were divided into an early and late cohort of 30 patients and 23 patients respectively. These cohorts covered the first 2 years and the final year of the study. All patients underwent endocrine assessment at defined time intervals. Patients with functional tumours were excluded. Variables reported included operative time, rate of postoperative CSF leak, rate of DI, degree of resection on MRI, endocrine assessment and visual assessment.

**Huang et al.** [11] reported on 273 patients who underwent endoscopic transsphenoidal pituitary adenoma resection between December 2014 and August 2021 at Shanghai Changzhgen Hospital. All procedures were performed by 3 neurosurgeons without the assistance of an ENT surgeon. Endocrine outcome is defined for functional adenomas at a defined interval, but not defined for non-functional adenomas. Cases were divided into an early, middle and late period of 91 cases each. Variables reported included operative time, rate of postoperative CSF leak, rate of DI, degree of resection on MRI scan and endocrine assessment. Visual outcome is not reported.

### Demographic findings

#### Patient demographics

Patient demographics are reported variably between studies. Variables reported included age, gender, type of pituitary adenoma, preoperative endocrinopathy, visual function and radiological factors. These are reported in Table 3.

**Table 3:** A table demonstrating the demographic and preoperative data for the included studies

	Mean age	Gender	Adenoma type	Endocrinopathy of FPA	Endocrinopathy of NFPA	Vision	Tumour size	Tumour invasion
Kenan et al.	41.3 group I 48.1 group II	Not reported	19/78 (24%) NFPA 59/78 (76%) FPA	28/59 (47%) prolactinoma 21/59 (35%) somatotropinoma 4/59 (7%) corticotropinoma 2/59 (3%) thyrotropinoma 4/59 (7%) mixed	Not reported	Not reported	Not reported	Not reported
Leach et al.	51^*^	70/125^*^ (56%) M 55/125^*^ (44%) F	73/114 (64%) NFPA 41/114 (36%) FPA	22/41 (53%) somatotropinoma 10/41 (24%) corticotropinoma 9/41 (22%) prolactinoma 6/114 (5%) PA	Not reported	61/125 (49%) VF in at least 1 eye	Macroadenoma 106/125^*^ (85%)	Not reported
Bokhari et al.	56.7	35/79 (44%) M 44/79 (56%) F	39/79 (49%) NFPA 40/79 (51%) FPA	19/40 (48%) somatotropinoma 16/40 (40%) prolactinoma 4/40 (10%) corticotropinoma 1/40 (2%) thryrotropinoma	Not reported. 22/79 (28%) hypopituitarism for all patients	22/79 (28%) VF abnormality 11/79 (14%) VA abnormality	72/79 (91%) macroadenomas 7/79 (9%) microadenomas	Not reported
Chi et al.	52.45 group I 49.25 group II	45/80 (56%) M 35/80 (44%) F	34/80 (43%) NFPA 46/80 (57%) FPA	26/46 (57%) prolactinoma 9/46 (20%) somatotropinoma 3/46 (6%) corticotropinoma 3/46 (6%) thyrotropinoma 5/46 (11%) mixed	Not reported	57/80 (72%) VF abnormality	64/80 (80%) macroadenoma 16/80 (20%) microadenoma	Not reported
Shou et al.	Not reported	78/178 (44%) M 100/178 (56%) F	92/178 (52%) NFPA 86/178 (48%) FPA	43/86 (50%) somatotropinoma 32/86 (37%) prolactinoma 4/86 (5%) corticotropinoma 7/86 (8%) mixed	Not reported	65/178 (^) VF abnormality 6/178 (^) CNO	Not reported	Hardy I 17/178(9.5%) Hardy II 58/178 (32.6%) Hardy III 72/178 (40.4%) Hardy IV 31/178 (17.4%)
Qureshi et al.	52.2 group I 52.7 group II	43/78 (57%) M 35/78 (43%) F	Not reported	Not reported	Not reported	57/78 (73%) VD	75/78 (96%) macroadenoma 3/78 (4%) microadenoma MTV 6.51cm^3^ group I 15.73cm^3^ group II	Not reported
Kim et al.	53	155/331 (46.8%) M 176/331 (53.2%) F	331/331 (100%) NFPA	Only reported NFPA	227/331 (68.6%) Hypopituitarism 127/331(^) GH deficiency 92/331 (^) ACTH deficiency 62/331 (^) TSH deficiency 197/331 (^) gonadotrophin deficiency 33/331 (10%) panhypopituitarism	167/322 (51.9%) VIS 1 107/322 (33.2%) VIS 2 25/322 (7.8%) VIS 3 23/322 (7.1%) VIS 4	283/331 (85.5%) macroadenoma 46/331 (13.9%) giant macroadenoma	45/331 (13.6%) Knosp 0 55/331 (16.6%) Knosp 1 87/331 (26.3%) Knosp 2 103/331 (31.1%) Knosp 3 41/331 (12.4%) Knosp 4
Younus et al.	52	305/600 (51%) M 295/600 (49%) F	441/600 (73%) NFPA 159/600 (27%) FPA	67/159 (42%) prolactinoma 53/159 (33%) somatotropinoma 38/159 (25%) corticotropinoma	Not reported	299/600 (^) VDef 13/600 (^) CNO	MMD: 23.3mm, 24.5mm, 22.5mm and 23.3mm got each quartile	Cavernous sinus invasion^#^: 28/150, 37/150, 44/150, 41/150.
Boetto et al.	59	30/53 (56%) M 23/53 (44%) F	53/53 NFPA	Only reported NFPA	Endocrine symptoms not defined: 5/30 (17%) | 5/23 (22%)	28/53 (52%) VDef	MMD 27.5mm MTV 7.37cm^3^	5/53 (9.4%) Knosp 0 12/53 (23%) Knosp 1 16/53 (30%) Knosp 2 8/53 (15%) Knosp 3a 3/53 (5.7%) Knosp 3b 9/53 (17%) Knosp 4
Huang et al.	52	137/273 (50.2%) M 136/273 (49.8%) F	182/273 (66%) NFPA 91/273 (34%) FPA	50/91 (56%) somatotropinoma 38/91 (42%) prolactinoma 2/91 (2%) corticotropinoma 1/91 (1%) thyrotropinoma	Not reported	124/273 (45%) VDef	6/273 (2%) microadenoma 231/273 (85%) macroadenoma 36/273 (13%) giant macroadenoma	17/273 (6.2%) Knosp 0 65/273 (24%) Knosp 1 89/273 (33%) Knosp 2 73/273 (27%) Knosp 3 29/273 (9.8%) Knosp 4

Absolute number reported if available, and then percentage of cohort

Abbreviation: *NFPA* non-functioning pituitary adenoma, *FPA* functioning pituitary adenoma, *PA* pituitary apoplexy, *VF* visual field, *VA* visual acuity, *CNO* cranial neuropathy of oculomotion, *VD* visual deficit including acuity, field or cranial neuropathy, *MTV* mean tumour volume, *VIS* visual impairment score, *VDef* visual deficit not specified, *MMD* mean maximal diameter

*Baseline cohort that included pathology other than pituitary adenomas. ^Percentages unable to be calculated as cohorts not presented discretely. ^#^Cavernous sinus invasion not defined

### Outcome findings

Outcome variables are reported inconsistently between studies. The variables are presented in Table 4 and summarised below.

**Table 4:** A table demonstrating the outcome variables for the included articles

	Rates of resection	Average operating time (min)	Postoperative CSF leak rate	Visual outcomes	Endocrine outcome/cure FPA	Endocrine outcome NFPA	Rate of DI	Further treatment
Kenan et al.	NFPA: 6/10 (55%)| 7/9 (77%) Macroprolactinoma: 6/11 (55%) | 9/12 (75%) Macrosomatotropinoma: 4/8 (50%) | 6/9 (66%) All macroadenomas*: 16/29 (55%) | 22/30 (73%)	Macroadenoma* 175 | 130 Microadenoma* 130| 95	1/40 (2.5%) | 1/38 (2.6%)	Not reported	Microadenomas were excluded Macroprolactinoma: 6/11 (55%) | 8/12 (66%) Macrosomatotropinoma: 4/8 (50%) | 6/9 (66%)	New anterior pituitary insufficiency: 2/40 (5%) | 0/38 (0%)	Permanent DI: 0/40 (0%) | 1/38 (2.6%)	Not reported
Leach et al.	Large tumour residual: 2/53 (4%) | 4/72 (6%)	NFPA* 120 | 91 FPA 137 | 145	4/125 (3.2%)	VF improvement*: 16/20 (80%) | 38/41 (93%) VF unchanged/worse: 4/20 (20%) | 3/41 (7%)	Somatotropinoma: 12/15 (80%) | 6/7 (86%) Corticotropinoma: 2/4 (50%) | 5/6 (83%)	New anterior hypopituitarism: 8/53 (17%) | 18/72 (25%)	New permanent DI 2/53 (4%) | 4/72 (6%)	Postoperative radiotherapy 37/125 (30%)
Bokhari et al.	Overall 50/79 (63%) GTR NFPA 19/39 (49%) GTR FPA 31/40 (78%) GTR: 56% | 58% | 77%	Not reported	Total 2/79 (3%)	VF 89% | 75% | 100%	Overall cure*: 15% | 41% | 78% Somatotropinoma 11/19 (58%) Prolactinoma 7/16 (44%) Corticotropinoma 2/4 (50%) Thyrotropinoma 1/1 (100%)	All patients eupituitary preop remained postop	Permanent DI 2/79 (2.5%)	34 (43%) required further treatment: 21/34 (62%) pharmacotherapy, 11/34 (32%) radiotherapy, 2/34 (6%) further surgery
Chi et al.	Overall GTR*: 21/40 (52.5%) | 30/40 (75%)	Not reported	Overall: 1/40 (2.5%) | 3/40 (7.5%)	VF improvement: 21/28 (75%) | 26/29 (89.7%)	Overall*: 7/19 (36.8%) | 18/27 (66.7%)	All patients eupituitary preop remained postop	Overall: 3/80 (3.25%)	Not reported
Shou et al.	Overall* 129/178 (72.5%) NFPA 66/92 (72%) FPA 63/86 (73%)	Not reported	1/178 (0.5%)	Not reported	Overall 38/86 (44%) Cure for invasive FPA: 2/19 (11%) | 10/35 (29%)	Not reported	Not reported	NFPA 5/92 (5.4%) salvage RTx 12/86 (14%) salvage RTx
Qureshi et al.	GTR: 8/9 (89%) | 64/68 (94%)	*206 | 164	1/9 (11%) | 0/68 (0%)	VF: 8/8 (100%) | 47/49 (95.9%)	Not reported	Panhypopituitarism: 1/9 (11%) | 9/68 (13%)	Permanent DI: 1/9 (11%) | 4/68 (5.8%)	Not reported
Kim et al.	GTR* 63% | 80.1%	Not reported	8/331 (2.4%)	Overall: 73.4% improvement, 2.7% worsened Predictor for VR OR 2.15 (1.25–3.70)	Only reported NFPA	All patients: normal to normal 18.7%, normalised 6.3%, improved 15.4%, persistent 27.2%, worsened 32.9% Predictor for ER OR 1.23 (0.65–2.32)	10/331 (3%)	Not reported
Younus et al.	GTR overall*: 83/150 (55%) | 102/150 (68%) | 105/150 (70%) | 118/150 (79%) GTR NFPA*: 61/109 (56%) | 85/113 (75%) | 72/110 (65%) | 97/109 (89%)	Not reported	5/150 (3%) | 2/150 (1.3%) | 1/150 (0.7%) | 1/150 (0.7%)	Normal vision postop: 139/150 (93%) | 132/150 (88%) | 125/150 (83%) | 133/150 (89%) Worsened vision postop: 0/150 (0%) | 2/150 (1%) | 5/150 (3%) | 0/150 (0%)	EC all FPA*: 68% | 78%| 88% | 90% EC prolactinoma: 74% | 80% | 93% | 89% EC somatotropinoma: 67% | 73% | 87% | 92% EC cortictropinoma: 57% | 80% | 80% | 91%	Eupituitary postop: 81% | 84% | 87% | 90%	Not reported	Not reported
Boetto et al.	GTR: 18/30 (60%) | 19/23 (83%)	*127 | 113	0 (0%) | 2/23 (8.6%)	Complete recovery: 8/30 (27%) | 10/23 (43%) Partial recovery: 9/30 (30%) | 3/23 (13%) Stabilisation: 13/30 (43%) | 10/23 (43%) Worsening: 0 (0%) | 0 (0%)	Only NFPA reported	Worsened hypopituitarism: 4/30 (13%) | 1/23 (4.3%)	Permanent DI: 1/30 (3.3%) | 1/23 (4.3%)	Not reported
Huang et al.	GTR overall*: 47/91 (51.6%) | 59/91 (64.8%) | 63/91 (69.2%)	*169 | 152| 147	11/91 (12.1%) | 6/91 (6.6%) | 5/91 (5.5%)	Not reported	EC overall: 12/32 (37.5%) | 16/27 (59.3%) | 18/32 (56.3%) EC prolactinoma: 5/15 (33.3%) | 5/7 (71.4%) | 7/16 (43.8%) EC somatotropinoma: 6/15 (40%) | 11/12 (55%) | 10/15 (66.7%)	Not reported	Permanent DI 4/273 (1.5%)	Not reported.

Reported as cohort: groups a | group b | group c etc. Number within each cohort and percentage reported. OR odds ratio (95% CI)

Abbreviation: *NFPA* non-functioning pituitary adenoma, *FPA* functioning pituitary adenoma, *PA* pituitary apoplexy, *VF* visual field, *VA* visual acuity, *CNO* cranial neuropathy of oculomotion, *VD* visual deficit including acuity, field or cranial neuropathy, *MTV* mean tumour volume, *VIS* visual impairment score, *VDef* visual deficit not specified, *MMD* mean maximal diameter, *ND* not documented, *VR* visual recovery, *ER* endocrine recovery

*A statistically significant difference between groups showing an improvement over time. ^A cohort where it is not clear if it is exclusively pituitary adenomas

**Rates of gross total resection** were reported in all 10 articles [2, 3, 7–9, 11, 12, 15, 17, 20]. Six of the articles [2, 9, 11, 12, 17, 20] demonstrate a significant improvement in the degree of resection in later cases compared to the earlier cases. Three of the articles [7, 8, 15] report an improvement, but it was not statistically significant. One article [3] commented on the rate of ‘large tumour residual’ between early and late surgical groups.

**Average operative time** was reported in 5 articles [2, 3, 7, 11, 15] where a statistically significant reduction in operating time occurs in later surgical groups compared to earlier groups. The remaining 5 articles [8, 9, 12, 17, 20] do not report the operative time.

**CSF leak rate** was reported in all 10 articles [2, 3, 7–9, 11, 12, 15, 17, 20]. CSF leak rate was only reported as an overall percentage in 4 articles [3, 8, 12, 17]. The remaining 6 articles [2, 7, 9, 11, 15, 20] reported CSF leak rate in each surgical group. Two articles [7, 9] demonstrated an increase in the rate of postoperative CSF leak from 2.5 to 7.5% and 0 to 8.6% respectively. The remaining 4 articles [2, 11, 15, 20] reported either stable CSF leak rates, or decreasing rates that were not statistically significant

**Visual outcomes** were reported in 7 articles [3, 7–9, 12, 15, 20]. Of these, 1 article [3] reported a statistically significant difference in the proportion of patients with visual field improvement between surgical groups. One other article [12] reported an OR 2.15 (1.25–3.7) for the effect surgical experience would have on visual recovery. The remaining 5 articles [7–9, 15, 20] reported a trend showing high rates of good visual outcomes in the late surgical groups compared to the earlier groups, or had high rates of visual improvement between all groups. There were 3 articles [2, 11, 17] that did not report visual outcomes.

**Endocrinological outcomes** were variably reported in all 10 articles [2, 3, 7–9, 11, 12, 15, 17, 20].

*Rates of endocrine outcome or cure for functional adenomas* were reported in 7 articles [2, 3, 8, 9, 11, 17, 20]. Of these articles, all reported a trend towards improving rates of endocrine cure between early and late surgical groups with 3 articles [8, 9, 20] demonstrating a statistically significant change. Of the remaining 3 articles, 2 [7, 12] articles only reported on non-functioning pituitary adenomas and 1 article [15] did not report endocrine cure outcomes.

*Rates of endocrine outcome or dysfunction for non-functional adenomas* were reported in 8 articles [2, 3, 7–9, 12, 15, 20]. Of these, 6 articles [2, 3, 7, 12, 15, 20] reported on how the rate of endocrine outcome changed between surgical groups. Three articles [2, 3, 7] report on the rate of new or worsened anterior pituitary insufficiency/hypopituitarism between early and late surgical groups. These outcomes are as follows: Kenan et al. 5 to 0%, Leach et al. 17 to 25%, Boetto et al. 13 to 4.3%. Two articles [8, 9] report that “all patients who were eupituitary preoperatively remained eupituitary postoperatively”. One article [20] reported on the rate of postoperative patients that were eupituitary between surgical groups, demonstrating a trend to improve rates over time. One article [15] reported the rate of new panhypopituitarism between surgical groups, 11% in the early group and 13% in the late surgical group. One article [12] examined all pituitary hormones postoperatively and reported the following: 18.7% eupituitary pre- and postoperatively, 6.3% normalised, 15.4% improved but not normalised, 27.2% unchanged, 32.9% worsened. This article also reported an OR 1.23 (0.65–2.32) for predicting if surgical experience affected endocrine outcomes. Two articles [11, 17] did not report endocrine outcomes for patients with non-functional adenomas.

*Rates of permanent diabetes insipidus* were reported in 8 articles [2, 3, 7–9, 11, 12, 15]. Of these articles, 4 articles [2, 3, 7, 15] report on how the rate of permanent DI changes between surgical groups. The remaining 4 articles [8, 9, 11, 12] report the overall rate of permanent DI. The rates are low ranging from 0 to 6% and therefore do not demonstrate any trends.

**Surgical technique** used was clearly defined in all articles [2, 3, 7–9, 11, 12, 15, 17, 20]. These techniques did not change during the study period.

### Study quality

Overall study quality was determined to be moderate in 3 articles [7, 9, 12] and low in 7 articles [2, 3, 8, 11, 15, 17, 20], these are demonstrated in Table 5. No articles were of high methodological quality. Common features between articles of low study quality included defining the method of quantitative visual assessment, defining tumour size and tumour invasiveness radiologically, defining the method of endocrine assessment for functioning and non-functioning adenomas, defining the timing interval for when visual assessment, endocrine assessment and postoperative imaging occurred, defining the complete hormonal function of non-functioning pituitary adenomas and reporting what further treatments if any were required after long-term follow-up.

**Table 5:** Quality assessment consensus table

Paper	Demographic details	Preoperative variables	Postoperative variables	Complications	Learning curve	Overall quality
Kenan et al.	Moderate	Low	Low	High	High	Low
Leach et al.	High	Low	Low	Low	Low	Low
Bokhari et al	High	Low	Low	High	High	Low
Chi et al.	High	Low	Moderate	High	High	Moderate
Shou et al.	Moderate	Low	Low	Low	High	Low
Qureshi et al.	High	Low	Low	High	High	Low
Kim et al.	High	High	Moderate	High	High	Moderate
Younus et al.	High	Low	Low	Poor	High	Low
Boetto et al.	High	Low	Moderate	High	High	Moderate
Huang et al.	High	Low	Low	High	High	Low

## Discussion

### Rates of gross total resection

Overall, this review demonstrates that 6 articles [2, 9, 11, 12, 17, 20] in the literature report a statistically significant improvement in the proportion of patients receiving a gross total resection with increased surgical. The remaining articles [7, 8, 15] demonstrated a trend towards improvement, but it was not significant. Based on these findings, gross total resection appears to be an outcome that follows a learning curve. This may be explained by surgeons becoming more comfortable attempting resection of residual disease adherent to neurovascular structures, or becoming more adept at using the endoscope to visualise tumour remaining in obscured regions of the operative field. It is not possible based on the variety of methodologies to determine in greater detail the relationship between surgical experience and degree of resection. This would be challenging to analyse given the heterogeneity of pituitary adenomas and the different surgical goals depending on the case.

### CSF leak rate

Overall, this review demonstrates no statistically significant association with rates of postoperative CSF leak as surgical experience increases. For the articles that did report CSF leak rate in each surgical group, there were 2 articles with increased rates in later surgical groups, and the other 4 articles reported stable or improved trends. It is not possible to make any recommendations given the small numbers of postoperative CSF leak rates between different articles. Another confounding factor when assessing this outcome is the variability amongst studies in who was performing the reconstruction and closure following tumour resection: in some studies, it was performed by neurosurgeons, in some by ENT surgeons and in one series was initially performed by ENT but than later began to be performed by the neurosurgeons. Previous literature has shown that the rate of postoperative CSF leak more broadly reduces over time as skull base teams gain experience [22].

### Visual outcome

Our review demonstrates 2 articles [3, 12] that show surgical experience significantly increases the chance of visual improvement and/or recovery. The remaining articles also demonstrate improvement, but it is not statistically significant. Due to the variability in how visual outcome is reported between articles, it is not possible to determine whether it improves with surgical experience.

### Endocrine outcome

Overall, our review demonstrates that endocrine outcomes are variably reported in the literature making direct comparisons between articles challenging. A surgical learning curve is easier to demonstrate for functional adenomas compared to non-functional adenomas. Three articles [8, 9, 20] demonstrate a statistically significant increase in the proportion of patients achieving an endocrine cure with increasing surgical experience.

Non-functional adenomas were reported less frequently in the literature. Only 1 article [12] examined all pituitary hormones postoperatively and reported whether patients improved, stabilised or worsened.

### Learning curve

This systematic review demonstrates that there are some outcome variables that do improve with surgical experience. These include degree of gross total resection, visual outcome and rate of endocrine cure for functional adenomas. It is not clear what drives these improvements, but may relate to increased surgeon confidence and aptitude in accessing more difficult-to-reach components of the tumour, thereby allowing more complete resection and potentiating decompression of the visual apparatus or removal of the hypersecreting adenoma. However, the way these articles examine surgical experience is not consistent and is largely based on arbitrary post hoc analysis. In addition, each outcome variable is not reported consistently between articles. This is most significant for outcomes such as endocrine function in non-functioning adenomas or visual outcomes.

Furthermore, it is important to note that the combination of each surgical team varied between articles. This may have affected the individual results of each article. Unfortunately, further analysis of this is not possible for the reasons stated earlier.

If more research is to be undertaken into understanding the learning curve for endoscopic pituitary surgery, a more rigorous and systematic approach to outcome reporting is required. This will enable accurate and translatable assessments of outcomes between articles. Characterisation of what outcomes have a longer learning curve may help focus training on particularly difficult components of the surgery. This training could be enhanced through the use of novel surgical training tools such as surgical simulation models [23].

We have developed an outcome reporting tool that we have implemented in our institutional skull base unit to facilitate systematic and standardised data collection about patients having pituitary surgery (refer to Table 6). The tool is pragmatic and easy to complete, but contains a variety of clinically significant variables.

**Table 6:** Proposed prospective data collection tool for patients undergoing endoscopic pituitary surgery. Data points entered are either dichotomous yes/no answers (previous radiotherapy), numerical grade or size (Knosp grade, maximal tumour diameter), multiple choice single answer (endocrine hormone function), or multiple choice multiple answer (reconstruction technique)

Preoperative		Intraoperative		Postoperative	
*Previous radiotherapy*	⊗ Yes ⊗ No	*CSF leak*	Graded as per Esposito et al 2007	*CSF leak (early = < 7 days, late = >7 days)*	⊗ Early ⊗ Late
*Endocrine*	Condition of each axis as determined by endocrinologist (Hypo/normo/hyper)	*Major vascular injury*	Defined as arterial injury requiring harvesting of muscle patch for management	*DI (temporary = no longer requiring DDAVP on first postoperative review)*	⊗ No ⊗ Temporary ⊗ New and permanent ⊗ Preexisting
*Radiological*	Maximal tumour diameter in any dimension	*Reconstruction (graded approach to repair, each number indicates escalating level of reconstruction)*	** 1. Onlay repair. local mucosa and/or dural substitute ** ** 2. Multilayer repair** *Inlay repair* ⊗ Fat ⊗ Dural substitute ⊗ Fascia ⊗ Cartilage/bone *Onlay* ⊗ Fascia ⊗ Dural substitute ⊗ Fat *Vascularised* ⊗ Nasoseptal ⊗ Inferior turbinate ⊗ Lateral turbinate ** 3. Packing** ⊗ Gelfoam ⊗ Nasopore ⊗ BIPP ** 4. Sealant** ⊗ Yes ⊗ No ** 5. Preop lumbar drain** ⊗ Yes ⊗ No	*Surgical goal achieved*	⊗ Yes ⊗ No
	Knosp grade				
*Previous surgery*	⊗ Yes ⊗ No			*Visual outcome at 6–12 weeks*	Visual acuity
	Number of previous surgeries				If possible link copy of OCTs/visual fields. If not, describe change in VF defect
					⊗ Worsened ⊗ Stable ⊗ Improved
*Visual status (quantitative measure)*	⊗ Visual acuity ⊗ If possible link copy of OCTs/visual fields. If not, describe VF defect			*Endocrine outcome* (**new deficit** = new insufficiency that requires treatment, **recovery of deficit** = preop deficit that no longer requires supplementation, **endocirne cure** = complete endocrine remission, **partial endocrine cure** = improved medical control but still requiring treatment)	Condition of each axis as determined by endocrinologist (Hypo/normo/hyper)
					⊗ No change ⊗ New deficit ⊗ Recovery of deficit ⊗ Endocrine cure ⊗ Partial endocrine cure
*Surgical goal*	⊗ Optic decompression ⊗ Hormonal control ⊗ Gross total resection			*Extent of resection (GTR = no residual tumour on first postoperative imaging)*	⊗ GTR ⊗ Not GTR
				*Condition at last known follow-up*	⊗ No recurrence ⊗ Residual under surveillance ⊗ Recurrence under surveillance ⊗ Progression of residual/recurrence requiring further treatment ⊗ Recurrence of endocrinopathy requiring further treatment

The formatting of certain words being bolded is to function as a minor subheading within the table cell

### Limitations

There are several limitations to this review. The main limitation is that there was significant variation in the outcomes that studies reported, and heterogeneity in the way each outcome was measured. This precluded meta-analysis that could quantitatively assess the learning curve and how it impacted individual outcome measures. Included studies were retrospective in nature with the attendant bias related to the choice of the outcome measures after the outcomes had occurred.

## Conclusions

We have demonstrated that a learning curve exists for some outcome variables for endoscopic pituitary surgery. However, there is significant heterogeneity in the current body of literature which makes clear comparisons difficult. If more research is to be undertaken to better define factors involved in shaping the learning curve for endoscopic pituitary surgery, we would recommend a rigorous and systematic approach to outcome reporting. Prospective observational studies may be the best study design to investigate this learning curve. Better defining this surgical curve will help improve patient safety by allowing more targeted and efficient training for surgical trainees.

### Supplementary information

ESM 1 (PDF 1190 kb)

## Data Availability

Not applicable
